# Strategies to improve hidden curriculum in nursing and medical education: a scoping review

**DOI:** 10.1186/s12909-023-04652-z

**Published:** 2023-09-11

**Authors:** Amin Hosseini, Elham Ghasemi, Alireza Nikbakht Nasrabadi, Leila Sayadi

**Affiliations:** 1 School of Nursing and Midwifery, Department of Medical Surgical Nursing, Tehran University of Medical Sciences, Tehran, Iran; 2 Community-Based Participatory Research Center, Tehran University of Medical Sciences, Tehran, Iran; 3 School of Nursing and Midwifery, Department of Medical Surgical Nursing, Tehran University of Medical Sciences, Tehran, Iran; 4 School of Nursing and Midwifery, Tehran University of Medical Sciences, Tehran, Iran; Nursing and Midwifery Care Research Center, School of Nursing and Midwifery, Tehran University of Medical Sciences, Tehran, Iran

**Keywords:** Hidden curriculum, Nursing education, Medical education, Scoping review

## Abstract

**Background:**

The importance of hidden curriculum cannot be neglected in education. Despite much research in the field, there have been limited studies on HC improvement in nursing and medical education. This scoping review aimed to determine the scope of strategies to improve HC in nursing and medical education.

**Method:**

PubMed, EBSCO/Cumulative Index to Nursing and Allied Health Literature (CINAHL), Cochrane Library, Scopus, Web of Science, Proquest and Persian-language databases of Magiran and SID were searched in January 2023 without a time filter. According to the PRISMA flow diagram, two independent reviewers selected the records that fit the inclusion and exclusion criteria via title and abstract screening. Next, the reviewers studied the full texts of the related articles. The data extracted from the selected articles were tabulated and ultimately synthesized.

**Findings:**

Out of the eight examined studies, published from 2017 to 2022, only one was in the field of nursing and seven were in medicine. The central strategies were implementing new curricula to replace the previous ones, utilizing team-based clinical clerkship, proposing a HC improvement model, implementation a case-based faculty development workshop, implementation longitudinal and comprehensive educational courses, and incorporating an educational activity into a small group program.

**Conclusion:**

Students and faculty members familiarization on the topic of HC, implementing new curricula, utilizing team-based clerkship, and using comprehensive models were among the HC improvement strategies. Focusing on upgrading the learning environment, particularly the clinical settings, can also be helpful in HC improvement.

## Introduction

The hidden curriculum (HC) is a crucial form of curriculum consisting of what is beyond determined formal and informal curricula. The HC is unofficial but can lead to learning [1, 2]. In another definition, the HC refers to unspoken expectations, unintended learning outcomes, implicit messages, and a student-created curriculum [3] representing knowledge, ideas, perceptions, performance, and values that students gain beyond their official lessons [4]. It can lead to stable and powerful educational outcomes [5].

The HC in medical education takes on greater importance considering the professional nature, various educational aspects, and extensive learning settings, particularly the clinical settings [3]. In other words, values, beliefs, and normal behaviors in medical sciences are conveyed through the HC [6]. This is also an evident, crucial case in nursing. Teaching professional nursing behavior according to the curricula influences the quality of provided nursing care. However, it has always been a challenging task. The challenges are not solely limited to the content or skills that need to be taught, but also associated with teachers and the method and time of education. They also encompass various aspects related to teachers, teaching methods, and the timing of education Moreover, a wide range of both positive and negative attitudes and behaviors at the clinical environment, as a relatively uncontrollable setting, and the dominant clinical culture can pose problems for nursing and medical education, thereby highlighting the role of the HC [7–9]. In clinical and other learning environment, the HC is often in contrast with the formal curriculum. Students usually pay attention to the differences, especially when the gap is related to ethical and professional issues. In such settings, students tend to internalize behaviors and values modeled by their instructors and peers during their academic years [5].

The HC can have positive and negative outcomes. Some of the positive outcomes include development of critical thinking skills, active choices from the surrounding environment, lifelong learning, cultural awareness, professional socialization, professional identity, professional ethics and status, and the link of theory and practice [5]. Some of the negative outcomes of the HC are reinforcement of negative competitiveness, reinforcement of obedience over initiative and suppression of innovation, learner maladjustment and social isolation, disinterest, inflexibility and being deprived of different experiences, student dependence on the faculty, influence on professional identity, low self-esteem, acceptance of the dominance of formal curriculum, unwillingness to collaborate, and reduced levels of activity, ability, and confidence [3, 5]. These, in turn, can affect interactions with patients and successful clinical reasoning [5].

Negative outcomes rising from inconsistency between the HC and official curriculum can lead to concerns in education [5], which need to be effectively addressed. Considering the importance of the HC improvement, it is necessary to conduct more research in this field [7]. Studies on the HC have focused on professional socialization of students in the clinical environment, professional identity and ethics, and communication with patients [10–12]. In Iran, most studies have adopted a qualitative approach to examine the experience of nursing and medical students with the HC [13, 14]. However, there has been limited studies on HC improvement [6, 15] as the central topics have been improving the learning environment, tackling the negative effects of the HC, identifying the positive and negative influences of the HC, facilitating HC learning, and the influences of the HC in nursing and medical education [8, 16–20]. Since the scoping review is a type of secondary study used to synthesize research evidence obtained from original research studies, it is a suitable option among the methods of review studies when a researcher seeks to find answers to questions such as “what” and “why” in the field of a specific topic. Scoping reviews are particularly useful when the main subject of the research and the available documents related to it have not been widely and comprehensively investigated [21]. Therefore, scoping review was used in this study.

The HC can bolster or undermine the objectives of the formal curriculum. Accordingly, shaping or enhancing hidden curricula, rather than eliminating them, need closer attention in order to maximize their constructive results [22]. Studies show that inconsistencies between the HC and the formal curriculum have negative outcomes to the education process. Therefore, it is necessary to search for strategies to improve the HC To this end, it seems necessary to conduct studies that can delineate the scope of previous research on the HC and determine the literature gap. This scoping review aimed to determine the scope of strategies to improve HC in nursing and medical education.

## Method

This was a scoping review, a type of secondary research to synthesize evidence from existing literature. It is particularly used when the main research topic and available related materials have not been extensively and comprehensively examined [21]. This scoping review utilized the Arksey and O’Malley Framework that proposes five essential stages: (1) Identifying the research question, (2) Identifying the relevant literature, (3) Selecting studies, (4) Charting the data, (5) Collating, summarizing, and reporting the results [21, 23].

### Identifying the research question

The guiding research question was posed as follows: What strategies have been proposed to improve HC in nursing and medical education?

### Identifying the relevant literature

PubMed, EBSCO/Cumulative Index to Nursing and Allied Health Literature (CINAHL), Cochrane Library, Scopus, Web of Science, Proquest and Persian-language databases of Magiran and SID were searched on January, 2023. The keywords were determined based on the texts and consultation with experts in the field. The search strategy was set according to each database. The keywords were placed together in the search using AND/OR Boolean operators. For example the search strategy adopted for PubMed was as follows:

(nursing OR nurs* OR medicine OR medical) AND (“hidden curriculum” OR “implicit curriculum” OR “informal curriculum” OR “hidden curricula” OR “implicit curricula” OR “informal curricula”).

The keywords were selected from MeSH terms, among others: “nursing, nurs*, medicine and medical”.

Primary studies on the topic of HC in nursing or medical education that focused on strategies for HC improvement were reviewed. Studies with a variety of quantitative, qualitative and mixed method approaches contains interventional (RCT, semi-experimental, etc.) and non-interventional (cross-sectional, cohort, etc.) designs were included in the study. The population inclusion criteria were students and the faculty. Studies on topics other than the HC, on other topics than HC improvement, and in other fields than medicine and nursing were removed from this scoping review. Reviews, case studies, editorials, conference papers, and theses were also excluded.

### Selecting studies

**Fig. 1:**
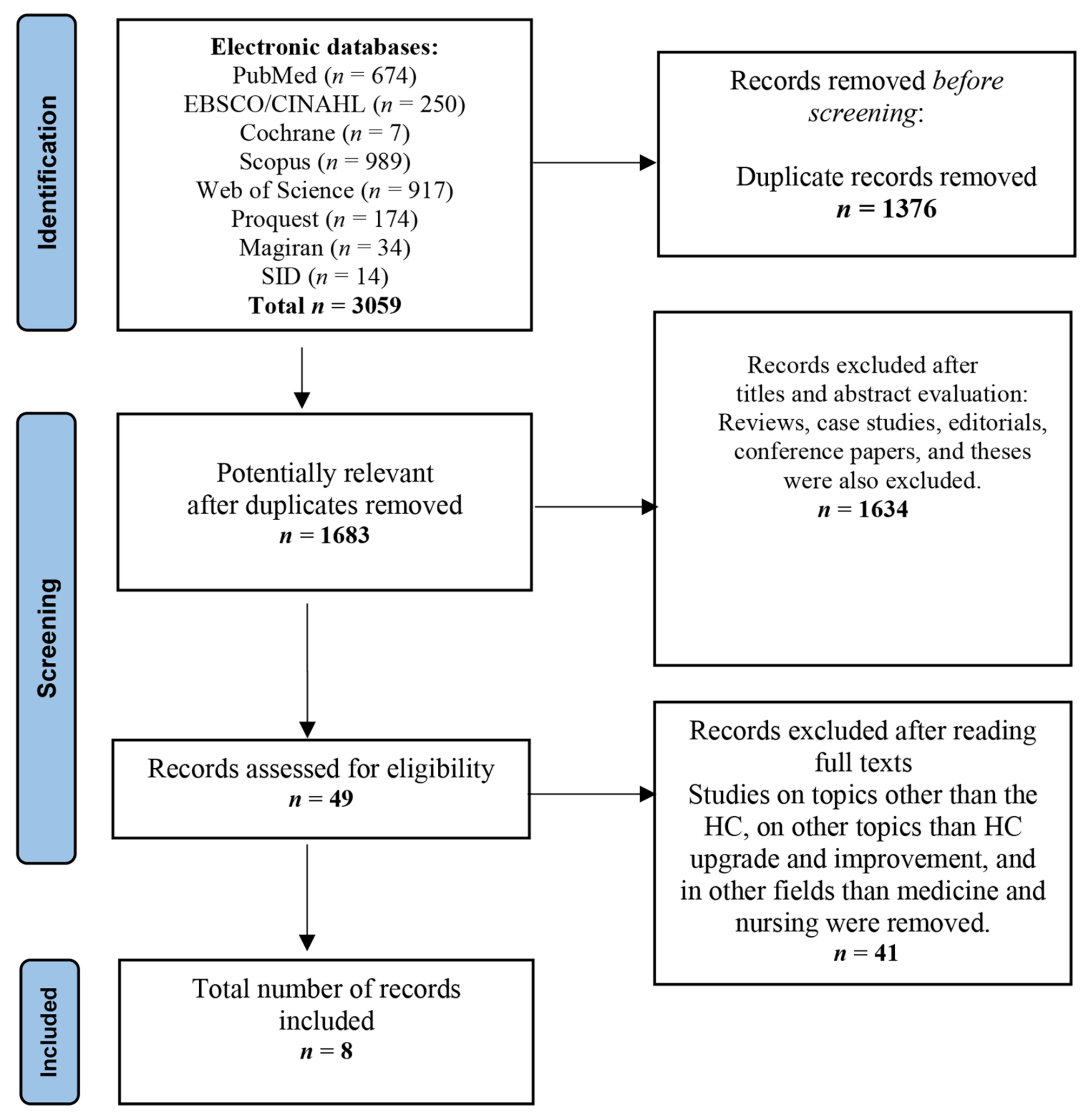
PRISMA flow diagram for the study selection process

There was no restriction on the type of input studies. The retrieved studies were imported into EndNote and duplicates were excluded. First, two reviewers independently selected the records that fit the inclusion and exclusion criteria via title and abstract screening. Next, the reviewers studied the full texts of the selected articles. In such cases any disagreements resolved by either consensus. EndNote was used to manage the references. The PRISMA flow diagram was used to demonstrate the selection process [24] (Fig. 1).

### Charting the data

The data extraction form including authors’ information, publication year, study objective, target population and sample size, study type, and important results was prepared as a table. The two reviewers completed the form for each study independently. In such cases any disagreements resolved by either consensus.

### Collating, summarizing, and reporting the results

At this stage, the two reviewers summarized the data in terms of geographical location of the studies, methodologies, and strategies for HC improvement, reporting the results in a table. A scoping study will need some analytic framework or thematic construction in order to present a narrative account of existing literature [21]. Due to the heterogeneity of the data, studies were combined to summarize descriptive findings, followed by a textual narrative synthesis.

## Results

As shown on the PRISMA flow diagram (Fig. 1), a total of 3059 studies were imported into EndNote from different databases. Once duplicates were removed (n = 1376), title and abstract screening was conducted on 1683 studies and the full texts of 49 studies were examined. Finally, eight articles were selected. Out of the selected studies, which were published from 2017 to 2022, four articles were set in the US [18–20, 25], two in Iran [16, 26], one in England [8], and one in Lebanon [17]. One study was in the field of nursing [16] and seven were in medicine [8, 17–20, 25, 26]. Four studies were longitudinal [17, 18, 20, 25], two were quasi-experimental [16, 20], one was qualitative [8], and one was a model design [26]. Totally, five studies were on students [8, 17–19, 25]. One study implemented an intervention and assessed the faculty [20]; another study implemented an intervention on the faculty and evaluated a group of students [16]. One of the selected studies proposed a model for HC improvement in medical education [26]. The specifications of the articles are summarized separately in Table 1.

**Table 1:** Summary of the studies included in the present scoping review

Authors, publication year, country	Objective	Target population and demographic data	Study type	Intervention & Data gathering	Findings
Keshtgar et al., 2017, Iran [16]	Determining the influence of HC management on professionalism	Nursing students, n = 69 Age mean = 20.58 gender = 63.8% female, 36.2% male	Quasi-experimental	Six-hour workshop on professional ethics focusing on existing strengths conveyed through the HC / The data obtained from professional behavior questionnaire	Professional ethics workshop for instructors and the faculty improved the average of professional behavior score in students. Indirect emphasis on learning adherence to ethics, commitment, and responsibility of providing care for patients. The post-intervention mean scores of professional behavior in nursing, midwifery, and emergency medicine students increased significantly. Nursing: 60.12 ± 0.70 vs. 67.11 ± 13.10 (pre vs. post test), P < 0.0001. Midwifery: 50.22 ± 8.75 vs. 56.14 ± 12.16 (pre vs. post test), P < 0.0001. emergency medicine: 40.23 ± 9.87 vs. 50 ± 10.14 (pre vs. post test), P < 0.0001.
Yazdani et al., 2019, Iran [26]	Designing a HC management model in medical education	N/A	Model design	N/A	Emphasis on environmental and human factors, formal curriculum, learner’s influenceability filter, management of knowledge and learning by managing the HC in medical education
Zgheib et al., 2020, Lebanon [17]	Determining the influence of the new curriculum in improving wellbeing and the learning environment, increasing compassion among students, and tackling the negative effects of the HC during a seven-year period	Medical students, n = 58	Longitudinal	Implementing a new seven-year patient-centered curriculum / The perceived learning environment was assessed by the Dundee Ready Education Environment Measurement survey and the hidden curriculum was examined using a locally developed survey.	Improving students’ perception and responses to the HC / Emphasis on the implementation of a curricular intervention by accurate design based on educational theories, methods, and standards. The aspects of medical students’ perceptions and counter responses to the HC improved in the study. fourth-year medical students (mean: 70), first- and second-year students (mean: 60, 56) p = 0.038.
Chapa et al., 2020, United States [18]	Determining the influence of the new curriculum in upgrading the learning environment in undergraduate medical education with the aim of HC management	Medical students, n = 71	Longitudinal	Implementing a new curriculum with focus on distance learning content such as podcasts and online group discussions between students and the faculty / students completed an online self-assessment questionnaire regarding their awareness/experiences.	Improving interpersonal relationships and connection in learners, improving the learning environment from students’ view, and the success of the curriculum in students’ development from the faculty’s view. 98% of the participants believed that implementing a new curriculum improved the learning environment in their general medicine program.
Shelton and Campo-Engelstein, 2021, United States [25]	Description of virtue ethics as a strategy to tackle the challenges and unfavorable effects of the HC in undergraduate medical education	Medical students, n = 182	Longitudinal	N/A	Description of virtue ethics course in medical education as an approach to address and amend the unfavorable effects of the HC and reinforcing students’ knowledge of positive elements in the learning environment / Emphasis on critical perspective in the absence of ideal clinical models
Henschen et al., 2020, United States [19]	Comparison of the effect of team-based and supervised individual-based clerkship on improving learning environment, team-based attitudes, perceptions of encountering the negative outcomes of the HC, communication with the patient, and professional efficacy	Medical students, n = 329	Longitudinal	Clinical clerkship for medical students based on two methods: team-based method contain completing clerkship for upper and lower year students and supervised individual-based / Students complete survey assessing the hidden curriculum; and the Attitudes Toward Health Care Teams (ATHCT) scale.	Obtaining a better mean of perceived support in encountering the potentially negative team-based effects of HC with regard to caregiver duties. Medical students in the team-based clerkship group reported more perceived support than those in the supervised individual-based clerkship group when encountering the potentially negative effects of the HC. Mean: 4.6 vs. 4.3 (p = P < 0.001).
Neve and Collett, 2017, England [8]	Explaining students’ experiences of small group program intervention in identifying the positive and negative effects of the HC, facilitating HC learning and attention to its influence on the profession	Medical students, n = 34	Qualitative	N/A / The study used the wiki contributions from four Year-3 Jigsaw groups. A descriptive thematic analysis was performed.	Favorable effects of the program in identifying the positive and negative aspects of the HC, learning, and its effects / Encouraging students to think critically on the HC can empower them to make active choices about which messages they want to internalize.
Farid et al., 2022, United States [20]	Determining the effects of a faculty development workshop in managing the challenges of the HC with emphasis on quintuple goals revolving around the learning environment: 1. Analyzing the role of the faculty members to improve the learning environment 2. Identifying negative aspects of the learning environment critically 3. Rethinking the performance in creating an appropriate learning environment 4. Presenting the faculty’s strategies to confront the negative aspects of the learning environment 5. Committing to adopting a strategy to improve the learning environment in curriculum planning	Medical faculty members, n = 60 Age mean = 49 Sex = 75% female, 25% male	Quasi-experimental	Implementation a workshop for the faculty to empower them for present strategies and commitment to implement them in encountering the negative aspects of the learning environment / A preworkshop and postworkshop survey was used to assess the workshop.	Success of the majority of the participants in achieving the set goals / Emphasis on the role of workshops and seminars in education development to improve the learning environment. More than 70% of the faculty evaluated the method successful in all five goals regarding the learning environment.

Most existing studies centered around improving the learning environment or settings influenced by the formal and hidden curricula [17–20]. Others targeted the effects of HC management on professional behavior and efficacy [16, 19], tackling the negative effects of the HC [17, 19, 25], identifying the positive and negative effects of the HC, and facilitating learning about the HC and its influence in practice [8].

Five studies focused on making learners aware of the hidden curriculum. Neve and Collett [8] had explained the students’ experiences of implementating small group program regarding the identification of positive and negative effects of the hidden curriculum. Two studies tried to increase students’ awareness of the hidden curriculum by teaching effective physician-patient communication and group discussions between professors and students in the form of implementing new curricula [17, 18]. Yazdani et al. [26] also presented a factor called learner’s influenceability filter and considered it to be a very important factor in informing learners on the way to improve the hidden curriculum. This study also found human and environmental factors to be among the effective factors on the hidden curriculum. In the area of identifying the factors affecting the hidden curriculum, Farid et al. [20] implement a workshop to present improvement strategies and factors affecting the formation of this curriculum. Among improvement strategies, Keshtgar and Shelton highlighted the role of professional ethics in an interventional and descriptive approach [16, 25]. Among the studies, implementing clinical clerkships as a team and with supervision was another strategy to improve the hidden curriculum [19].

## Discussion

The results showed that HC improvement strategies in the reviewed studies included different forms of training such as development workshops in nursing and medicine, longitudinal and comprehensive training courses, and small-scale group program, new curricular design, implementation, and assessment, team-based clinical clerkship, and a model proposed for medical education.

Two studies suggested that teaching students and faculty and conducting implementation workshops for faculty members can improve the HC. The workshops can improve the learning environment, which is influenced by the formal and hidden curricula, and lead to the faculty’s correct understanding of their roles and responsibilities [16, 20]. Workshops allow discussions on the HC, its influence on the learning environment, and strategies for its improvement through case-based examination among the faculty. Workshops also provide a space for the participants to discuss shared experiences and present implementable solutions [20]. In this regard, In a study that was held in the form of a workshop, presenting the experiences of the participants regarding the hidden curriculum determined that this concept is effective to improve professional development [27].

Another strategy of HC improvement was small-scale group programs and training courses [8]. Shelton and Campo-Engelstein [25] suggest a virtue ethics course in medical education as an approach to address and amend the unfavorable effects of the HC and reinforce students’ knowledge of positive elements in the learning environment. Longitudinal training courses during the academic years can immensely help students to prepare for entering the medical practice, creating numerous learning opportunities. A study on medical students in England showed that courses focusing on practical teaching, small groups and speeches including treatment, and skills of documentation and management of critical clinical responsibilities significantly increased the post-course reported self-confidence scores of students [25]. Training courses can boost students’ critical thinking skills to recognize and to encounter the negative effects of the HC. Shelton and Campo-Engelstein emphasize the prominence of a critical perspective in the absence of ideal clinical role models when tackling the unfavorable outcomes of the HC Training courses give students the chance to prepare themselves gradually to accept their future responsibilities. By recognizing educational shortcomings, these courses are aimed to reform the processes and empower learners for the future. They can encourage students to critically reflect about their learning experiences. However, it is crucial to also note the positive aspects of students’ experiences, reinforcing which can substantially contribute to HC management [28].

Developing new curricula to replace old curricula is another strategy for improving the HC in nursing and medical education. Two studies have utilized this strategy to improve the learning environment and address the negative effects of the HC [17, 18]. The implementation of a new curriculum focused on students’ professional skills development has led to positive results. For example, Crigger and Godfrey (2014) introduced a framework for professional identity formation and professional ethics, and the long-term implementation of this framework resulted in professional development [29]. Dwinnell and Barley (2000) incorporated discussions on medical students’ daily clerkship events and faculty feedback into the curriculum, which proved to be effective in developing students’ professional skills. Transforming or reforming old curricula to meet professional requirements can be a suitable strategy for improving the HC. It is important for the approach to shift from a faculty-centered approach to student-centered programs, such as implementing group discussions, providing feedback, and fostering communication based on experiences. This shift increases the likelihood of success [30].

In the model proposed by Yazdani et al. [26]. (2019), several components were highlighted for improving the HC in medical education These components include environmental and human factors, management of knowledge and learning, and the learner’s influenceability filter [26]. Refining the formal curricula and what is taught to students is considered a crucial component of HC improvement. The Accelerating Change in Medical Education consortium-wide meeting in 2015 recommended the development of comprehensive, integrated, and standardized formal curricula to address the challenges of the healthcare system. Value-based care and improving the health system and public health were suggested for clinical sciences curricula [31].

Additionally, team-based primary care clerkship and utilizing teams to receive perceived support have been reported to be influential in addressing the potentially negative effects of the HC [19]. Peers in a team play a substantial role in learning how to encounter the HC. Near-peer learning experiences have been found to have a wide range of advantages for learners [32] allowing for more inter-student communication and direct conveyance of experiences. Team-based learning can contribute significantly to conveying educational content and messages in both the clinical environment and the classroom [33].

Scoping review showed that teaching about the HC in different forms, team-based clerkship, and comprehensive models were among the strategies used for HC improvement in medical and nursing education. Course-based teaching seems more influential in terms of further stability and persistence of learning. However, it should be noted that designing new curricula and continuity in the long term can also be listed among other HC improvement strategies [34]. Given that learning and clinical environment can be considered similar in medicine and nursing, these approaches can be used separately or together for HC improvement, particularly in nursing. The focal point in the strategies discussed by the studies was the learning environment. Accordingly, improving the learning environment in these two disciplines, especially nursing, can remarkably reduce the negative results of the HC. It seems that since much learning takes place clinically at the hospitals, improving the environment can be more influential for students [35]. Standardizing and accrediting hospitals, focusing on training and retraining the workforce in hospitals, and implementing professional commitment and behavior models in hospitals can be greatly helpful in this respect. Additionally, teaching approaches, as emphasized in most studies, can be adopted for students and the faculty combined with new curricula and team-based methods [36].

There are limited studies on improving the HC in medical and nursing education, possibly due to the difficulty of conducting studies in this field. Changing behaviors, values, and interactions of learners and teachers requires long-term action and accurate preparation and planning. It is also possible that such actions have been carried out in medical and nursing schools and educational centers, but not reported in the form of studies. Another reason for limited studies might be the hidden and unidentifiable or uncontrollable nature of the topic. However, the fact that selected studies on strategies for HC improvement were published from 2017 to 2022 might indicate that researchers have taken up the topic of HC improvement in recent years, paving the way for more studies in the coming years. Out of the eight selected studies, seven were in the field of medicine and only one was in nursing, indicating that the above reasons are more serious in nursing education.

### Strengths and limitations

To the authors’ knowledge, this is the first scoping review on HC improvement in nursing and medical education. Using a methodological framework to guide the review helped conduct the stages more transparently and accurately. Despite the authors’ attempt to set up a comprehensive search strategy, a limited number of studies were included in the review. Focusing on electronic databases without searching the gray literature because of research process conditions was a limitation in this study. Another limitation was lack of assessment of the methodological quality of the studies.

## Conclusion

Students and faculty members familiarization on the HC, implementing new curricula, utilizing team-based clerkship, and using comprehensive models were among the HC improvement strategies, mostly focusing on upgrading and improving the learning environment. These strategies could be integrated into students’ education separately or mixed. Given the clinical nature of medicine and nursing, strategies suggested for medical students could also be helpful for nursing students. Focusing on improving the learning environment, particularly the clinical settings, can also be helpful in HC improvement.

## Data Availability

The datasets used and/or analyzed during the current study are available from the corresponding author on reasonable request.
